# Evaluation of Antibacterial Effects of an Oral Rinse Containing Ocimum tenuiflorum and Ocimum gratissimum on Streptococcus mutans and Lactobacillus Species

**DOI:** 10.7759/cureus.67975

**Published:** 2024-08-27

**Authors:** Akshetha Loganathan, Remmiya Mary Varghese, Aravind Kumar Subramanian, Rajeshkumar Shanmugam

**Affiliations:** 1 Orthodontics and Dentofacial Orthopedics, Saveetha Dental College and Hospitals, Saveetha Institute of Medical and Technical Sciences, Saveetha University, Chennai, IND; 2 Nanobiomedicine, Saveetha Dental College and Hospitals, Saveetha Institute of Medical and Technical Sciences, Saveetha University, Chennai, IND

**Keywords:** lactobacillus spp, antibacterial activity, oral rinse, herbal formulation, ocimum gratissimum, ocimum tenuiflorum, streptococcus mutans, dental caries

## Abstract

Background and aim: Dental caries, primarily caused by *Streptococcus mutans* and Lactobacillus spp., is a major global health issue. There is a growing need for effective, natural antimicrobial treatments. *Ocimum tenuiflorum* and *Ocimum gratissimum* are known for their medicinal properties, including antimicrobial activity. This study investigates the antibacterial efficacy of a herbal oral rinse derived from these plants. This study aimed to evaluate the antibacterial activity of an herbal formulation-based oral rinse prepared from *Ocimum tenuiflorum *and *Ocimum gratissimum *against *Streptococcus mutans* and Lactobacillus spp.

Methods: Fresh leaves of *Ocimum tenuiflorum *and *Ocimum gratissimum *were shade-dried, powdered, and extracted in distilled water. The extract was incorporated into an oral rinse formulation. The antibacterial activity was assessed using the agar well diffusion method, protein leakage and cytoplasmic leakage assays, and time-kill curve analysis. A commercial oral rinse was used as a standard.

Results: The herbal oral rinse exhibited significant antibacterial activity against both *Streptococcus mutans *and Lactobacillus spp. The zones of inhibition for *Streptococcus mutans *were 10 mm, 13 mm, and 15 mm at concentrations of 25 µg/mL, 50 µg/mL, and 100 µg/mL, respectively. For Lactobacillus spp., the inhibition zones were 10 mm, 12 mm, and 14 mm at the same concentrations. The protein leakage and cytoplasmic leakage analysis supported these findings, demonstrating the formulation's efficacy at low concentrations. Time-kill curve assays showed rapid bactericidal action, particularly at higher concentrations.

Conclusion: The *Ocimum tenuiflorum* and *Ocimum gratissimum-*based herbal oral rinse demonstrates strong antibacterial activity against key oral pathogens, suggesting that it could be a natural alternative to conventional oral rinses.

## Introduction

Dental caries, affecting approximately 2.5 billion people worldwide, is a major global health issue and the most prevalent untreated condition, with a 14.6% rise over the past decade, particularly impacting socially disadvantaged groups [1]. The prevalence varies by region, with higher rates in low- and middle-income countries due to factors such as high sugar intake, inadequate fluoride exposure, and poor oral hygiene. Vulnerable populations with low incomes, limited education, and restricted access to dental care are disproportionately affected [2]. The WHO emphasizes the need for preventive measures and integration of oral healthcare into primary health systems, aiming for universal health coverage for oral health by 2030. Comprehensive efforts to improve preventive care, dietary habits, and oral hygiene practices are essential to reduce dental caries prevalence and enhance global oral health [3].

Previous studies have investigated various interventions to reduce dental caries prevalence. A pilot study in Iran found that comprehensive community-based interventions using diverse educational methods and messengers, such as dentists and social media, were most effective in reducing early childhood caries among 24-month-old children [4]. In China, a study assessed dental caries prevention knowledge and attitudes among university hospital patients, revealing that while most had accurate knowledge and positive attitudes, effective implementation of dental health education programs is still needed [5]. In the United States, researchers compared simple versus complex caries prevention treatments in rural elementary schools using various analytical methods to assess non-inferiority and cost-effectiveness [6]. Additionally, a meta-analysis on xylitol's effectiveness in caries prevention indicated a lower preventive fraction compared to previous studies, attributed to the variety of xylitol products included [7]. These findings suggest that while community-based interventions, preventive dentistry education, and xylitol products show promise in reducing dental caries, further research is necessary to identify the most effective and cost-efficient strategies [8].

Herbal formulation-based products present a promising and cost-effective strategy for dental caries prevention due to several key factors. They are more accessible and economical than conventional treatments, enabling self-care practices without needing prescriptions or professional interventions, making them particularly beneficial in low-resource settings [9]. Many herbal formulations have natural antibacterial and anti-inflammatory properties that are effective against primary caries-causing bacteria like *Streptococcus mutans*, with studies showing significant reductions in bacterial counts and plaque formation [10]. Clinical trials have reported positive outcomes regarding the efficacy of herbal products in preventing dental caries, despite varying methodological quality. The integration of traditional knowledge with evidence-based medicine has led to a growing acceptance of herbal remedies, allowing a holistic approach to oral health that complements conventional treatments [11,12]. Thus, incorporating herbal formulations into dental care strategies offers a viable path for effective and accessible caries prevention, demanding further research to optimize their application.

In the present study, a herbal formulation was prepared using extracts from *Ocimum tenuiflorum* and *Ocimum gratissimum* leaves. This formulation was then used to create an oral rinse, which was tested for its antibacterial activity against *Streptococcus mutans* and *Lactobacillus spp*.

## Materials and methods

Preparation of *Ocimum tenuiflorum *and *Ocimum gratissimum *herbal formulation

**Figure 1:**
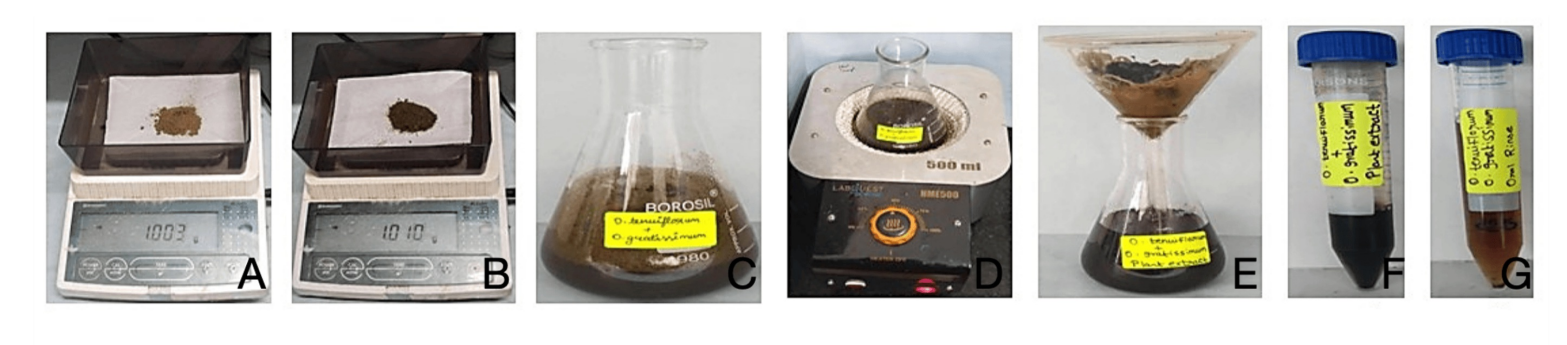
Preparation of herbal formulation-based oral rinse. The images show (A) *Ocimum tenuiflorum* powder, (B) *Ocimum gratissimum* powder, (C) addition of both powders in 100 g distilled water, (D) boiled using heating mantle, (E) filtration process, (F) filtered herbal formulation, and (G) *Ocimum tenuiflorum* and *Ocimum gratissimum* based oral rinse.

Fresh leaves of *Ocimum tenuiflorum *and *Ocimum gratissimum *were collected and thoroughly washed with distilled water to remove surface contaminants. The leaves were then shade-dried at room temperature until completely dehydrated. Once dried, the leaves were finely powdered using a mechanical grinder. A solution was prepared by combining 1 g of each powdered leaf with 100 mL of distilled water. This mixture was heated at 60°C for 15-20 minutes using a heating mantle. After boiling, the mixture was filtered gradually using filter paper (Figures 1A-1G).

Preparation of *Ocimum tenuiflorum* and *Ocimum gratissimum* herbal formulation-based oral rinse

The *Ocimum tenuiflorum* and *Ocimum gratissimum *herbal formulation-based oral rinse was prepared by combining 0.3 g of sucrose, 0.1 g of sodium lauryl sulfate, 0.001 g of sodium benzoate, and 500 µL of herbal formulation in 10 mL of distilled water. The sucrose acted as a sweetener, sodium lauryl sulfate as a foaming agent, and sodium benzoate as a preservative. The mixture was thoroughly mixed to create a herbal formulation-based oral rinse [11].

Antibacterial activity: agar well diffusion technique

The antibacterial activity of the *Ocimum tenuiflorum* and *Ocimum gratissimum *herbal formulation-based oral rinse was evaluated using the agar well diffusion technique against *S. mutans* and Lactobacillus spp. A standardized inoculum of *S. mutans*, Lactobacillus spp. (approximately: 106 CFU/mL) was evenly spread onto the surface of sterile Mueller Hinton agar plates. Wells of 9 mm diameter were punched into the agar using a sterile cork borer, and 100 µL of the herbal formulation-based oral rinse at concentrations of 25 µg/mL, 50 µg/mL, and 100 µg/mL were added into the respective wells. A commercial oral rinse was used as a standard for comparison. The plates were incubated at 37°C for 24 h, and the zones of inhibition around the wells were measured in millimeters [12].

Protein leakage analysis

Protein leakage from *S. mutans* and Lactobacillus spp. cells was assessed to determine the antibacterial efficacy of the herbal formulation-based oral rinse. The cells were treated with the herbal formulation-based oral rinse at concentrations of 25 µg/mL, 50 µg/mL, and 100 µg/mL, as well as a commercial oral rinse (standard) and untreated control. After treatment, the samples were centrifuged, and the supernatant was collected. The protein content in the supernatant was quantified by measuring the optical density at 280 nm using a UV-Vis spectrophotometer, indicating the extent of protein leakage from the cells.

Cytoplasmic leakage analysis

The cytoplasmic leakage was analyzed to evaluate the membrane-disruptive action of the herbal formulation-based oral rinse against *S. mutans* and Lactobacillus spp. The bacterial pathogens were treated with the herbal formulation-based oral rinse at different concentrations (25 µg/mL, 50 µg/mL, and 100 µg/mL), along with a commercial oral rinse and an untreated control. After incubation, the treated samples were centrifuged, and the supernatant was collected. The optical density of the supernatant was measured at 260 nm using a UV-Vis spectrophotometer to determine the release of cytoplasmic contents, reflecting cell membrane damage.

Time-kill curve assay

The time-kill curve assay was conducted to evaluate the rate at which the herbal formulation killed *S. mutans* and Lactobacillus spp. over a 5-h period. An inoculum of both oral pathogens standardized to approximately 106 CFU/mL was treated with the herbal formulation-based oral rinse at concentrations of 25 µg/mL, 50 µg/mL, and 100 µg/mL, alongside a commercial oral rinse (standard) and untreated control. Aliquots were taken at specific time intervals (0, 1, 2, 3, 4, and 5 h), serially diluted, and their optical density was measured using an ELISA plate reader at 600 nm. This method allowed for the plotting of time-kill curves to compare the effectiveness of the herbal formulation concentrations, the commercial oral rinse, and the untreated control in reducing *S. mutans *and Lactobacillus spp. over time [13].

## Results

Agar well diffusion technique

**Figure 2:**
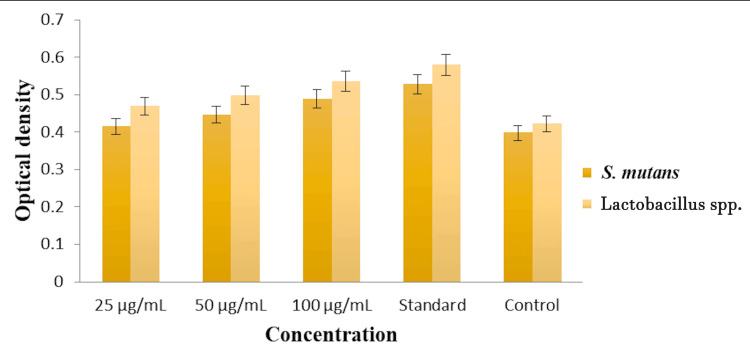
Protein leakage analysis of herbal formulation-based oral rinse against two different oral pathogens.

The antimicrobial activity of the *Ocimum gratissimum* and *Ocimum tenuiflorum* herbal formulation-based oral rinse was evaluated using the agar well diffusion technique against the oral pathogens *Streptococcus mutans* and Lactobacillus spp., with commercial oral rinse used as the standard for comparison. The zones of inhibition were measured in mm at concentrations of 25 µg/mL, 50 µg/mL, and 100 µg/mL. For *Streptococcus mutans*, the herbal formulation exhibited zones of inhibition of approximately 10 mm at 25 µg/mL, 13 mm at 50 µg/mL, and 15 mm at 100 µg/mL, compared to 19 mm for the standard. Similarly, for Lactobacillus spp., the zones of inhibition were around 10 mm at 25 µg/mL, 12 mm at 50 µg/mL, and 14 mm at 100 µg/mL, with the standard showing an inhibition zone of 18 mm. These results indicate that the *Ocimum gratissimum* and *Ocimum tenuiflorum* herbal formulation-based oral rinse demonstrate significant antimicrobial activity against both tested pathogens, with increasing effectiveness at higher concentrations. Figure 2 shows the standard commercial oral rinse displayed the highest zones of inhibition, underscoring its potent antimicrobial properties.

Cytoplasmic leakage analysis

**Figure 3:**
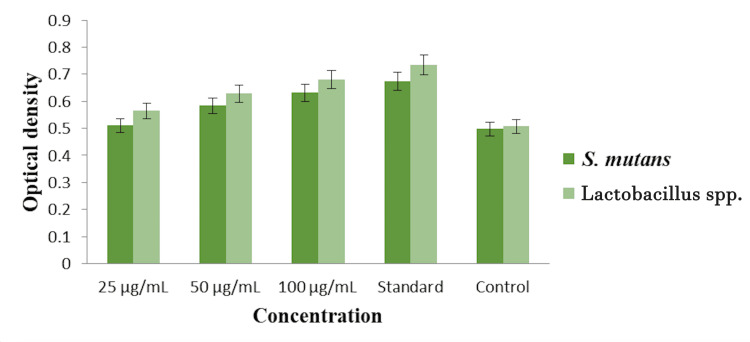
Cytoplasmic leakage analysis of herbal formulation-based oral rinse against two different oral pathogens.

The cytoplasmic leakage analysis of the *Ocimum gratissimum *and *Ocimum tenuiflorum *herbal formulation-based oral rinse was evaluated using optical density measurements at 260 nm against *Streptococcus mutans* and Lactobacillus spp., with a commercial oral rinse as the standard and untreated pathogens as the control. The optical density values indicate the extent of cytoplasmic leakage at different concentrations (25 µg/mL, 50 µg/mL, and 100 µg/mL) of the herbal formulation-based oral rinse. For *Streptococcus mutans*, the optical density was approximately 0.45 at 25 µg/mL, 0.5 at 50 µg/mL, and 0.55 at 100 µg/mL. The standard commercial oral rinse showed an optical density of around 0.65, while the control (untreated pathogens) had an optical density of about 0.4. For Lactobacillus spp., the optical density was around 0.5 at 25 µg/mL, 0.55 at 50 µg/mL, and 0.6 at 100 µg/mL. The standard commercial oral rinse exhibited an optical density of approximately 0.7, while the control showed an optical density of about 0.45. These results suggest that the *Ocimum gratissimum *and *Ocimum tenuiflorum *herbal formulation-based oral rinse induces cytoplasmic leakage in both *Streptococcus mutans* and Lactobacillus spp., with increasing leakage observed at higher concentrations. The commercial oral rinse demonstrated higher cytoplasmic leakage compared to the herbal formulation-based oral rinse, indicating its strong effectiveness. The control groups showed the lowest optical density, confirming the absence of cytoplasmic leakage without treatment (Figure 3).

Protein leakage analysis

The protein leakage analysis aimed to evaluate the efficacy of *Ocimum tenuiflorum *and *Ocimum gratissimum *herbal formulation as an oral rinse against *S. mutans *and Lactobacillus spp. with optical density measurements at concentrations of 25 µg/mL, 50 µg/mL, and 100 µg/mL compared to a commercial oral rinse (standard) and untreated pathogens (control). At 25 µg/mL, the optical densities were approximately 0.41 for *S. mutans* and 0.44 for Lactobacillus spp. indicating significant protein leakage relative to the control. Increasing the concentration to 50 µg/mL resulted in optical densities of 0.47 for *S. mutans *and 0.49 for Lactobacillus spp. showing enhanced efficacy. At the highest concentration of 100 µg/mL, the optical densities were 0.52 for *S. mutans *and 0.54 for Lactobacillus spp. marking the peak protein leakage and demonstrating the potent antimicrobial activity of the herbal formulation. The commercial oral rinse showed similar optical densities, with 0.54 for *S. mutans *and 0.56 for Lactobacillus spp., comparable to the herbal formulation at its highest concentration. The control group, representing untreated pathogens, had the lowest optical densities of approximately 0.35 for *S. mutans *and 0.38 for Lactobacillus spp., indicating minimal protein leakage without treatment. Overall, the herbal formulation displayed significant antimicrobial activity in a concentration-dependent manner, with its highest concentration performing equally with the commercial oral rinse, suggesting its potential as an effective natural alternative for oral hygiene as depicted in Figure 2.

## Discussion

The present study examined the antimicrobial efficacy of an herbal formulation-based oral rinse containing *Ocimum tenuiflorum *and *Ocimum gratissimum* against key oral pathogens, including *Streptococcus mutans* and Lactobacillus spp. The results demonstrated significant antibacterial activities of the formulation, with a concentration-dependent increase in efficacy. The zones of inhibition for *S. mutans *were approximately 10 mm, 13 mm, and 15 mm at concentrations of 25 µg/mL, 50 µg/mL, and 100 µg/mL, respectively, compared to 19 mm for the standard commercial oral rinse. Similarly, for Lactobacillus spp., the zones of inhibition were 10 mm, 12 mm, and 14 mm at the same concentrations, compared to 18 mm for the commercial rinse. The cytoplasmic leakage and protein leakage analyses further confirmed the formulation's antimicrobial properties, with increasing leakage observed at higher concentrations, indicating effective disruption of bacterial cell integrity.

The observed antimicrobial activity can be attributed to the bioactive compounds present in *Ocimum tenuiflorum* and *Ocimum gratissimum*, such as eugenol and thymol. These compounds are known for their potent antibacterial and antifungal properties [13]. Eugenol can disrupt the integrity of bacterial cell membranes, leading to cell death. The time-kill curve assay confirmed the rapid action of these compounds, with significant reductions in pathogen counts within the first four hours of exposure [14]. The concentration-dependent increase in the zones of inhibition as well as the extent of cytoplasmic and protein leakage further supports the hypothesis that these bioactive compounds are effective in disrupting microbial cell membranes and intracellular processes [15].

In recent years, there has been a growing interest in exploring the antibacterial properties of herbal formulations derived from *Ocimum tenuiflorum *and *Ocimum gratissimum *against various oral pathogens. Studies have shown that these herbal formulations exhibit significant antimicrobial activities against key oral pathogens such as *Streptococcus mutans *and Lactobacillus spp. [16,17]. The use of *Ocimum gratissimum *extract has also been found to be effective in reducing the effects and complications of opportunistic infections caused by bacteria like *Escherichia coli*, *S. aureus*, and *Pseudomonas aeruginosa *in HIV/AIDS patients [18]. Furthermore, research has highlighted the potential of Ocimum species, including *Ocimum basilicum*, *Ocimum kilimandscharicum*, *Ocimum gratissimum*, *Ocimum canum*, and *Ocimum tenuiflorum*, in inhibiting the growth of pathogenic bacteria [19,20]. These studies have demonstrated the antibacterial and antifungal potentials of essential oils and extracts derived from these Ocimum species against a range of microorganisms.

Moreover, the synthesized silver nanoparticles and zinc oxide nanoparticles using *Ocimum tenuiflorum* and *Ocimum gratissimum *herbal formulations have shown promising antibacterial activities against oral pathogens [21,22]. The silver nanoparticles derived from *Ocimum gratissimum *have also exhibited cytoplasmic and protein leakage effects on wound pathogens, further emphasizing their antibacterial properties [23]. In the context of oral health, herbal mouth rinses formulated with herbal extracts have been evaluated for their antimicrobial efficacy against salivary *Streptococcus mutans* and Lactobacillus spp. The results have indicated that herbal mouth rinses can effectively reduce the salivary *Streptococcus mutans* load, presenting a potential alternative to chemical mouthwashes like chlorhexidine [24].

Implications and applications

The results of this study suggest that the *Ocimum tenuiflorum *and *Ocimum gratissimum *herbal formulation could serve as a cost-effective and natural alternative to conventional oral rinses. This is particularly relevant in low-resource settings where access to commercial oral care products may be limited. The formulation's efficacy against multiple oral pathogens, including *S. mutans *and Lactobacillus spp., indicates its potential to reduce the prevalence of dental caries and other oral infections. Moreover, the natural origin of the formulation minimizes the risk of adverse effects associated with synthetic antimicrobials, making it a safer option for long-term use. The significant cytoplasmic and protein leakage observed in treated pathogens suggests that the formulation could also help in managing biofilms, which are often resistant to conventional treatments.

Strengths and limitations

One of the strengths of the present study is the comprehensive analysis of the antimicrobial activity of the herbal formulation using multiple assays. This approach provides a robust evaluation of the formulation efficacy and the mechanisms underlying its antimicrobial properties. Additionally, the concentration-dependent increase in efficacy highlights the potential for dose optimization in clinical applications. However, there are several limitations to our study. The in vitro nature of the experiments may not fully replicate in vivo conditions, and the lack of clinical trials means that the safety and efficacy of the formulation in human subjects remain to be validated. Furthermore, the variability in the concentration of bioactive compounds due to environmental factors could affect the reproducibility of the results. Future studies should address these limitations by conducting in vivo experiments and clinical trials to confirm the herbal formulation-based oral rinse efficacy and safety in human subjects.

Overall, the research on the antibacterial activity of *Ocimum tenuiflorum* and *Ocimum gratissimum *herbal formulations based oral rinse against oral pathogens, particularly *Streptococcus mutans *and Lactobacillus spp., underscores the potential of these herbal remedies in combating oral infections. The diverse studies reviewed provide different perceptions of the antimicrobial properties of Ocimum species and their applications in oral health and infectious disease management.

## Conclusions

The present study demonstrated the significant antimicrobial activity of a herbal formulation-based oral rinse derived from *Ocimum tenuiflorum *and *Ocimum gratissimum *against key oral pathogens, including *Streptococcus mutans* and Lactobacillus spp. The formulation exhibited a concentration-dependent increase in efficacy, with significant zones of inhibition and substantial cytoplasmic and protein leakage observed in treated pathogens. These findings suggest that the herbal formulation could serve as a natural and cost-effective alternative to conventional oral rinses, particularly in low-resource settings. However, further research, including clinical trials, is necessary to validate these findings and explore the formulation's potential in real-world applications. Our study provides a strong foundation for future research and development of natural oral care products, highlighting the potential of herbal formulations in improving oral health.
